# Scale-dependent landscape variables and linear infrastructures influence smooth newt (*Lissotriton vulgaris*) abundance in wetlands of a heavily urbanized lake

**DOI:** 10.1038/s41598-025-97988-z

**Published:** 2025-04-22

**Authors:** Boglárka Mészáros, József Bürgés, Mónika Tamás, Blanka Gál, Judit Vörös, Andrew J. Hamer, Dénes Schmera

**Affiliations:** 1 HUN-REN Balaton Limnological Research Institute, Klebelsberg Kuno street 3, Tihany, H-8237 Hungary; 2 National Multidisciplinary Laboratory for Climate Change, HUN-REN Balaton Limnological Research Institute, Klebelsberg Kuno street 3, Tihany, H-8237 Hungary; 3 Limnology Research Group, Center of Natural Sciences, University of Pannonia, Egyetem u. 10, Veszprém, 8200 Hungary; 4 Institute of Aquatic Ecology, HUN-REN Centre for Ecological Research, Karolina út. 29, Budapest, 1113 Hungary; 5 National Multidisciplinary Laboratory for Climate Change, HUN-REN Centre for Ecological Research, Karolina út. 29, Budapest, 1113 Hungary

**Keywords:** Habitat loss, Fragmentation, Agricultural activities, Linear infrastructure, Amphibians, Conservation biology, Wetlands ecology

## Abstract

The degradation of freshwater ecosystems due to land use changes is one of the major driver of global biodiversity loss and amphibian declines with these impacts varying across different spatial scales. Our study aimed to assess how natural and human-modified land affects smooth newt (*Lissotriton vulgaris*) abundance in the surrounding waterbodies of Lake Balaton, a highly urbanized area. We conducted aquatic trap surveys at 32 wetland sites during the breeding season and quantified land cover within 250, 500, and 1000-m radius buffer zones. We hypothesized that urban land use, cropland, and proximity to roads and railways would negatively correlate with newt abundance, while wetlands areas (marshes, swamps, periodically flooded grasslands) and natural terrestrial habitats (grasslands, forests and woodlands) would positively correlate, with effects varying across spatial scales. N-mixture models were used to analyse survey data, estimating abundance and examining relationships with covariates. Results revealed that wetland cover within a 500-m buffer zone increased newt abundance, probably due to supporting metapopulation connectivity. In contrast, cropland cover within 250 m and proximity to roads and railways negatively affected newt abundance. Conservation efforts should prioritize providing smooth newts with adequate breeding habitats and reducing disturbances from croplands, roads, and railways.

**Supplementary Information:**

The online version contains supplementary material available at 10.1038/s41598-025-97988-z.

## Introduction

The primary drivers of global biodiversity loss are the destruction and degradation of natural ecosystems caused by land use alterations, which often lead to habitat loss and fragmentation in terrestrial habitats^1,2^. Haddad et al.^3^ conducted a synthesis of 35 years of fragmentation experiments across various spatial scales. They found that habitat loss and fragmentation have caused a 13 to 75% reduction in biodiversity across five continents. More than 70% of the world’s remaining forests are now situated near modified environments. Freshwater wetlands, critical for biodiversity and essential ecosystem services such as fish nursery habitats and floodwater retention, face similar threats and are among the most endangered ecosystems^4^. It is estimated that 70% of the world’s wetlands have been destroyed or significantly compromised due to human activities^5–7^. Understanding the patterns and processes that determine how species respond to landscape changes in terrestrial and aquatic habitats is crucial in ecological research. This knowledge is key to understanding the relationship between biodiversity loss and degraded ecosystems, which is essential for developing effective management strategies in fragmented landscapes.

As wetland-dependent organisms, amphibians are particularly vulnerable to land-use change, which is one of the major drivers of global amphibian declines^8^. 40.7% of amphibian species are now threatened by habitat loss, fragmentation, and declining habitat quality due to plant harvesting, mining, pollution, urbanization, infrastructure development, and agricultural expansion^9^. For example, altered urban conditions, such as elevated temperatures because of impervious surfaces^10^ and increased light, noise, and chemical pollution^11^ indirectly impact biodiversity, potentially limiting species persistence and adding complexity to the interconnected effects of urbanization on amphibian populations^12^. As urbanization intensifies, infrastructure development such as road and railway construction, presents significant challenges for amphibians, leading to direct mortality from traffic, habitat degradation, and pollution from runoff along transportation routes^13,14^. Similarly to urbanization and infrastructure development, agricultural expansion harms amphibians by degrading and modifying landscapes through pesticide and fertilizer contamination^15^.

Beyond the substantial biological, physical, and chemical changes in both terrestrial and aquatic ecosystems^16^, human-induced land use changes are reshaping habitat structures through fragmentation effects^17,18^. The expansion of impervious surfaces—such as roads, railways, buildings, and pavements—driven by urbanization and land conversion^19–22^, along with deforestation resulting from agricultural practices^19,23,24^, are primary drivers of these environmental transformations leading to habitat loss, fragmentation and reduced connectivity. Due to their limited dispersal abilities, amphibians encounter greater challenges as a result of this diminished connectivity^25,26^. These landscape changes also fragment amphibian habitats at larger spatial scales, ultimately reducing long-term population viability^27^ by severely disrupting metapopulation dynamics^28^. Amphibians rely on both aquatic and terrestrial habitats to complete their complex life cycles. Terrestrial habitats provide food and shelter during juvenile and adult stages, while aquatic habitats are essential for breeding, supporting reproductive adults, eggs, and larvae^29^. Ecological connectedness between breeding sites and surrounding terrestrial habitats is crucial for maintaining amphibian metapopulations. Barriers like roads, railways, agricultural lands and urban impervious surfaces can disrupt these movements, potentially affecting metapopulation dynamics and reducing regional population sizes^27,30,31^. For example, Hamer et al.^32^ found a negative correlation between amphibian larval abundance and both highway proximity and road coverage, while^28^ reported that road density and development negatively affected anuran species richness.

The spatial scale of land-use effects plays a key role in how land use influences amphibian presence, distribution and abundance. The intensity of impacts changes across different scales, and the strength of the effects often depends on the scale being examined^33^. Land use change due to agricultural activities may reduce the availability of terrestrial habitats at a local scale and fragment dispersal corridors at a larger scale^34^, while roads may impact amphibians locally through direct mortality or pollutant runoff, and at larger scales by isolating subpopulations^28^. The effects of drastic landscape changes due to human activity may ultimately lead to genetic substructuring of populations^35^. Understanding these scales can provide key insights into the underlying mechanisms, with localized effects typically linked to direct impacts on individual survival, while larger-scale effects often result in habitat fragmentation and reduced metapopulation connectivity. Effective conservation planning depends on understanding how land use changes affect animal populations at various spatial scales. Focusing on a single scale to identify landscape characteristics influencing populations can result in misleading predictions and miss critical landscape effects. This understanding is vital for predicting the impact of human-driven land use changes on amphibians and for identifying key conservation areas^36^.

Here, we investigated how natural landscapes, human-modified landscapes and linear infrastructures influence the abundance of smooth newts (*Lissotriton vulgaris*) across various spatial scales around a heavily urbanized freshwater lake in Central Europe. We hypothesized that, in the hinterland of a highly urbanized lake, human-modified landscapes and linear infrastructures would negatively affect newt populations, whereas natural landscapes would support newt abundance. Additionally, we expected these effects to vary across different buffer zone sizes, coinciding with the preferred dispersal distance of *L. vulgaris*.

Since wetlands are essential breeding sites for amphibians^37^, we predicted that there would be a positive relationship between the proportion of wetlands (i) and the size of waterbodies (ii) and newt abundance. Additionally, considering that surrounding terrestrial habitats play a crucial role in the complex life cycle of amphibians by providing food and shelter during their terrestrial phase^38,39^, we predicted there would also be a positive relationship between forest and grassland coverage (iii) and abundance. In contrast, based on prior research on amphibians^9^, we predicted there would be a negative relationship between human-modified landscapes, including urban (iv) and agricultural (v), and newt abundance. Following this, we predicted that newt abundance would decrease near linear infrastructures, such as high-traffic roads (vi) and railways (vii), due to direct mortality^13,40^, the indirect effects of pollution^14^ and habitat fragmentation^32^.

## Results

### Newt surveys

A total of 290 smooth newts were captured across two surveys at 32 sampling sites: 164 individuals (mean = 5.12; SD = 8.62; min = 0; max = 42) in the first survey, and 126 individuals (mean = 3.94; SD = 6.67; min = 0; max = 26) in the second survey. The number of newts captured at each site and in each survey is summarized in Table S1.

### Probability of detection

**Fig. 1:**
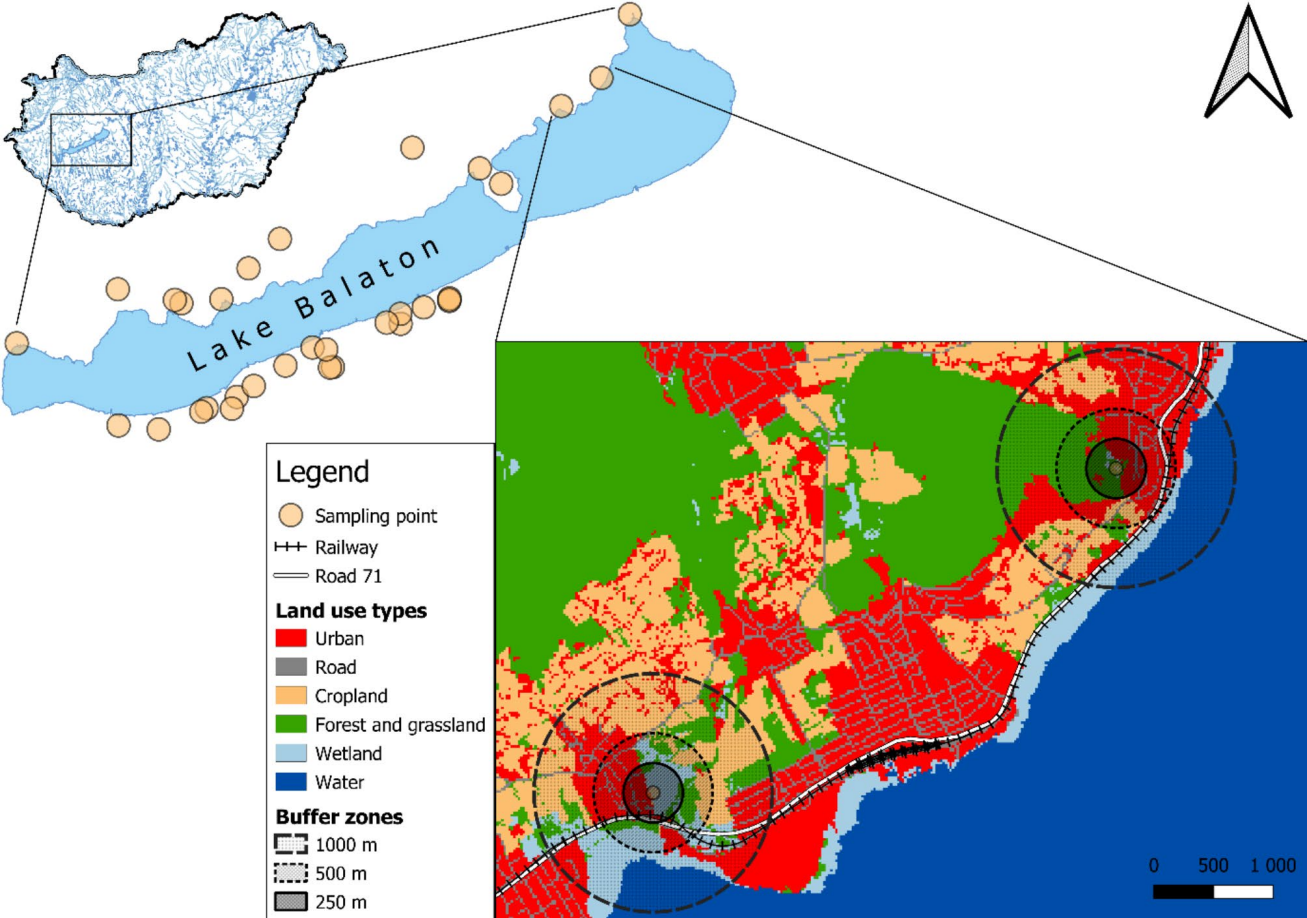
Map of Hungary (top-left) highlighting the study area, which includes the final 32 sampling points (center), along with two example sites illustrating buffer zones of different sizes. The names and WGS coordinates of the sampling points are provided in Supplementary Table S1. The figure was generated using QGIS version 3.28.4-Firenze^94^ (http://qgis.org) and the Ecosystem Map of Hungary^95^.

The detection sub-model including only water depth was the best-supported model (Table 1). This model revealed that the detection of smooth newts is negatively associated with water depth (Table 2), meaning that newts are more easily captured in shallower water. Accordingly, subsequent abundance models incorporated water depth as a detection covariate (Fig. 1).

**Table 1:** Model selection for detection covariates, based on the Watanabe-Akaike information criterion (WAIC).Models with a ΔWAIC < 2 were regarded as having the strongest support.

Model	Covariates		WAIC	ΔWAIC
**1**	**Intercept**	**DEPTH**	**282.32**	**0.00**
2	Intercept	DATE	299.09	16.77
3	Intercept	TRAPS	309.05	26.73
4	Intercept	WTEMP	310.53	28.20
5	Intercept	VEG	311.41	29.09

The best ranked model is shown in bold.

DEPTH = water depth; DATE = Julian date of survey; TRAPS = number of traps deployed at a site; WTEMP = water temperature; VEG = percentage of emergent vegetation.

**Table 2:** Summary of estimates of the posterior distribution for the best detection sub-model.

Model	Parameters	Mean	SD	2.5th	97.5th	f
1	Intercept	-1.518	0.412	-2.399	-0.776	
	**DEPTH**	**-0.604**	**0.160**	**-0.929**	**-0.305**	**100**

Estimates are provided with 95% Bayesian credible intervals and the proportion of the posterior distribution (*f*). Covariate relationships were considered important if their 95% Bayesian credible intervals did not overlap zero or if the proportion of the posterior distribution exceeded 90% (in bold).

### Model selection

Model selection results for the abundance sub-models analysing spatial-scale effects on smooth newt abundance indicated that the 500 m buffer zone was the most supported scale for urban land and wetland cover, while the 250 m buffer zone was optimal for cropland and terrestrial habitat cover (Table 3).

**Table 3:** Model selection for selecting the most appropriate Spatial scale.

Model	Covariates				WAIC	ΔWAIC
URBAN						
**1**	**Intercept**	**WATERBODY**	**NNDIST**	**URBAN250**	**281.88**	**0.77**
**2**	**Intercept**	**WATERBODY**	**NNDIST**	**URBAN500**	**281.11**	**0.00**
**3**	**Intercept**	**WATERBODY**	**NNDIST**	**URBAN1000**	**281.90**	**0.79**
CROPLAND						
**1**	**Intercept**	**WATERBODY**	**NNDIST**	**CROPLAND250**	**279.86**	**0.00**
**2**	**Intercept**	**WATERBODY**	**NNDIST**	**CROPLAND500**	**280.26**	**0.40**
3	Intercept	WATERBODY	NNDIST	CROPLAND1000	282.16	2.30
WETLAND						
1	Intercept	WATERBODY	NNDIST	WETLAND250	281.44	3.48
**2**	**Intercept**	**WATERBODY**	**NNDIST**	**WETLAND500**	**277.97**	**0.00**
**3**	**Intercept**	**WATERBODY**	**NNDIST**	**WETLAND1000**	**279.39**	**1.42**
TERRSITES						
**1**	**Intercept**	**WATERBODY**	**NNDIST**	**TERRSITES250**	**282.04**	**0.00**
**2**	**Intercept**	**WATERBODY**	**NNDIST**	**TERRSITES500**	**282.17**	**0.13**
**3**	**Intercept**	**WATERBODY**	**NNDIST**	**TERRSITES1000**	**282.18**	**0.14**

The best ranked models are shown in bold.

WATERBODY = waterbody size; NNDIST = nearest neighbour distance; URBAN250 = urban land cover in the 250 m buffer zone; URBAN500 = urban land cover in the 500 m buffer zone; URBAN1000 = urban land cover in the 1000 m buffer zone; CROPLAND250 = cropland cover in the 250 m buffer zone; CROPLAND500 = cropland cover in the 500 m buffer zone; CROPLAND1000 = cropland cover in the 1000 m buffer zone; WETLAND250 = wetland cover in the 250 m buffer zone; WETLAND500 = wetland cover in the 500 m buffer zone; WETLAND1000 = wetland cover in the 1000 m buffer zone; TERRSITES250 = Forest and grassland cover in the 250 m buffer zone; TERRSITE500 = Forest and grassland cover in the 500 m buffer zone; TERRSITES1000 = Forest and grassland cover in the 1000 m buffer zone;

Model selection results for the abundance sub-models examining landscape covariate effects indicated that smooth newt abundance was influenced by cropland and wetland cover, as well as by the proximity to linear infrastructures (Table 4).

**Table 4:** Model selection for the final N-mixture models.

Model	Covariates				WAIC	ΔWAIC
1	Intercept	WATERBODY	NNDIST	URBAN500	288.11	3.15
**2**	**Intercept**	**WATERBODY**	**NNDIST**	**CROPLAND250**	**279.86**	**1.90**
**3**	**Intercept**	**WATERBODY**	**NNDIST**	**WETLAND500**	**277.97**	**0.00**
4	Intercept	WATERBODY	NNDIST	TERRSITES250	282.04	4.07
**5**	**Intercept**	**WATERBODY**	**NNDIST**	**DISTROAD**	**278.84**	**0.87**
**6**	**Intercept**	**WATERBODY**	**NNDIST**	**DISTRAILWAY**	**278.61**	**0.65**

Models with the best support (ΔWAIC < 2) are highlighted in bold. The water depth (DEPTH) detection covariate was included in all models.

DISTROAD = Distance to road; DISTRAILWAY = Distance to railway.

### Relationships between newt abundance and landscape covariates

**Fig. 2:**
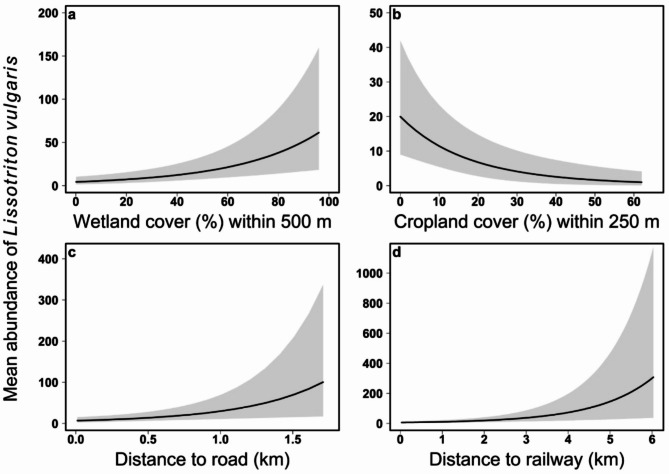
The relationship between the mean abundance of smooth newts (*L. vulgaris*) at a sampled waterbody and four environmental covariates (a-d). a: cropland cover (%) within 250 m; b: wetland cover (%) within 500 m; c: distance to a major road (km); and d: distance to a railway (km). The shaded areas represent the 95% Bayesian credible intervals, while the black line represents the mean estimated abundance of smooth newts.

Wetland coverage in the 500 m buffer zone increased the mean abundance of smooth newts, suggesting that higher wetland cover at an intermediate scale was associated with greater newt abundance (Table 5; Fig. 2a). Newt abundance was predicted to increase from no individuals at sites without wetland cover to over 30 individuals at sites with more than 80% wetland cover. In contrast, cropland cover in the 250 m buffer zone had a negative effect on the mean abundance of smooth newts, indicating lower newt abundance in areas with higher agricultural land cover near breeding sites (Table 5; Fig. 2b). Specifically, there was a predicted decline in abundance from 20 individuals at sites with no surrounding cropland, to fewer than 5 individuals at sites with more than 40% cropland cover. Furthermore, both the distance of main roads and railways were negatively associated with mean smooth newt abundance, indicating smaller populations to occur near high-traffic roads and railways (Table 5; Fig. 2c and d). There was a predicted increase in abundance from fewer than 5 individuals in the immediate vicinity of roads, to approximately 80 individuals at sites located more than 1.6 km from a major road. Additionally, newt abundance was predicted to increase from nearly zero individuals near railways to over 500 individuals at sites located more than 6 km from railway tracks.

**Table 5:** Summary of estimates of the posterior distribution for the best-supported abundance N-mixture models.

Model	Parameters	Mean	SD	2.5th	97.5th	f
1	Intercept	2.382	0.383	1.694	3.200	
	**WATERBODY**	**0.348**	**0.201**	**-0.044**	**0.744**	**95.89**
	**NNDIST**	**-0.743**	**0.255**	**-1.249**	**-0.248**	**100.00**
	**CROPLAND250**	**-0.958**	**0.303**	**-1.581**	**-0.394**	**100.00**
	Intercept	-1.619	0.435	-2.529	-0.840	
	**DEPTH**	**-0.575**	**0.160**	**-0.900**	**-0.272**	**100.00**
2	Intercept	2.417	0.369	1.758	3.207	
	WATERBODY	0.222	0.207	-0.182	0.629	85.88
	NNDIST	-0.176	0.222	-0.613	0.262	78.66
	**WETLAND500**	**0.727**	**0.201**	**0.335**	**1.125**	**100.00**
	Intercept	-1.621	0.417	-2.509	-0.873	
	**DEPTH**	**-0.648**	**0.162**	**-0.981**	**-0.343**	**100.00**
3	Intercept	2.430	0.369	1.765	3.216	
	**WATERBODY**	**0.375**	**0.206**	**-0.026**	**0.780**	**96.65**
	**NNDIST**	**-0.400**	**0.222**	**-0.834**	**0.034**	**96.47**
	**DISTROAD**	**0.563**	**0.201**	**0.171**	**0.961**	**100.00**
	Intercept	-1.603	0.419	-2.487	-0.842	
	**DEPTH**	**-0.630**	**0.161**	**-0.959**	**-0.327**	**100.00**
4	Intercept	2.468	0.363	1.812	3.236	
	**WATERBODY**	**0.387**	**0.203**	**-0.008**	**0.784**	**97.25**
	NNDIST	-0.206	0.213	-0.625	0.213	83.36
	**DISTRAILWAY**	**0.782**	**0.217**	**0.362**	**1.213**	**100.00**
	Intercept	-1.648	0.408	-2.494	-0.906	
	**DEPTH**	-0.667	0.159	-0.988	-0.367	100.00

Estimates are provided with 95% Bayesian credible intervals (representing the 2.5th and 97.5th percentiles of the posterior distribution) and the proportion of the posterior distribution (*f*). Covariate relationships were considered important if their 95% Bayesian credible intervals did not overlap zero or if the proportion of the posterior distribution exceeded 90% (in bold). Water depth (DEPTH) was included as a detection covariate in each model. The results of the unsupported models are summarized in Supplementary Table S4.

Mean abundance predictions for a given covariate were estimated within its observed range, from minimum to maximum, without extrapolating beyond these values.

## Discussion

Our study examined how natural and human-modified land influences the abundance of smooth newts at three different buffer sizes (250 m, 500 m, 1000 m) within the wetlands of a lake subjected to intensive urbanization and tourism activities. We predicted that urbanization, agricultural activities, and linear infrastructures like high-traffic roads and railways would negatively affect newt abundance. We also predicted that natural areas, such as wetlands (potential breeding sites), forests, and grasslands (terrestrial habitats), would support greater abundance. Additionally, we assumed that these effects would have significant impacts across different spatial scales. We used N-mixture models to estimate mean smooth newt abundance based on landscape covariates at different spatial scales. To ensure model stability and avoid convergence issues, we employed single-covariate detection models. Given our relatively small sample size (32 sites), adding multiple covariates could have led to statistical challenges and unstable estimates. Thus, we adopted a parsimonious approach, prioritizing reliability and interpretability.

As predicted, the best-fit models showed that the abundance of smooth newts was most strongly influenced by the amount of potential wetlands in the intermediate and large buffer zones (500 m and 1000 m). (Table 5; Fig. 2a). This suggests that smooth newts are more abundant in areas with more potential breeding sites. The impact occurs at the metapopulation scale, rather than directly influencing individual newts. Smooth newt populations are commonly structured as networks of spatially organized populations (SSPs) or metapopulations, utilizing distinct breeding habitat patches that are linked by dispersing individuals^41^. As a result, variations in occupancy and/or abundance arise from the extinction and colonization processes of metapopulations, ensuring stability even when local reproductive success varies^42,43^. Our finding aligns with previous studies demonstrating that connectivity between potential breeding sites is a critical factor in the metapopulation dynamics of this species. For example, Joly et al.^44^ discovered a positive correlation between the abundance of palmate (*Triturus helveticus*), alpine (*T. alpestris*) and crested (*T. cristatus*) newt species and the number of ponds within a 400-meter circular area. Moreover, it has been shown that connectivity, assessed by the number of ponds near the focal pond, and the area of the waterbody were both positively associated with *L. vulgaris* abundance^42^. Similarly, Falaschi et al.^41^ found that breeding site characteristics, particularly wetlands, were the primary determinants of smooth newt abundance dynamics. Based on our findings and previous studies, we propose that greater wetland coverage, which offers a higher number of potential breeding sites, enhances metapopulation connectivity and stability^45^. In addition to supporting our finding, we discovered that the size of the waterbody also positively correlated with the abundance of smooth newts. This can be attributed to the fact that larger breeding sites provide more extensive habitats and resources. These support larger populations, reduce fluctuations, and enhance survival, fecundity, carrying capacity, and overall vital rates^46,47^.

Our second key finding revealed that cropland coverage in the immediate and intermediate surroundings of breeding sites (250 m and 500 m) negatively affected newt abundance (Table 5; Fig. 2b), in line with previous studies. For example, Suárez et al.^48^ observed that anuran richness was lower in landscapes with greater agricultural development and less forest cover, based on anuran call survey data from 120 sampling points. Similarly, Curado et al.^24^ found that the disappearance of ponds in northern France over three decades was linked to a decrease in grassland and an increase in arable fields around the ponds. In our study, we found that the negative impact of agricultural land cover was most pronounced within a very close range (250 m) of the breeding habitats. This indicates a direct influence on individuals, likely due to contaminants from nearby agricultural activities. To meet the demands of the exponentially growing human population^49^, the use of agrochemicals, including herbicides, insecticides, pesticides, fungicides and fertilizers, has become a fundamental component of global agricultural systems, resulting in their widespread dispersion into the environment^15^. These agricultural chemicals are increasingly recognized as contributing to amphibian declines by disrupting developmental processes, altering sexual differentiation and increasing susceptibility to diseases, and also through negative effects on community interactions^50^. For instance, it has been shown that the presence of the yellow-legged frog (*Rana muscosa*) at ponds has been significantly reduced by pesticides^51^. Agricultural activities can also indirectly impact ecosystems by increasing eutrophication in aquatic habitats. This leads to greater algal growth and a higher density of snail hosts that may carry the trematode parasite *Ribeiroia ondatrae*, a pathogen harmful to amphibians^52^. In conclusion, these findings highlight the harmful effects of agricultural activities, particularly in areas near vulnerable wetlands that serve as breeding sites for amphibians. Proximity to cropland restricts amphibians’ movement, limiting their ability to colonize ponds and disrupting metapopulation connectivity^44,53^. As a result, the number of adult newts at these ponds decreases. These effects underscore the urgent need for more sustainable agricultural practices to protect these vital freshwater ecosystems.

Although a recent study in Finland by Vehkaoja et al.^39^ found that residential buildings negatively affect the occurrence and abundance of smooth newts, our results showed that natural terrestrial habitats and urban land use, including built-up areas with impervious surfaces, were not among the top-ranked models. However, numerous studies have highlighted the negative effects of urban land cover on other amphibian species. For example, Green et al.^54^ discovered that the proportion of impervious cover within 250 m of a wetland had the most significant negative impact on Pacific chorus frog (*Pseudacris regilla*) occupancy, while a study along an urban-rural gradient in Shanghai found that urban density significantly reduced overall anuran abundance and diversity^22^. Additionally, Woodford and Meyer^55^ observed that increased shoreline development density reduced the quality of breeding habitats, leading to a decline in the abundance of adult green frogs (*Rana clamitans melanota*) in northern Wisconsin lakes. Similarly, studies on southern two-lined salamanders (*Eurycea cirrigera*) have shown that larval abundance decreased with higher impervious surface cover in the upstream catchment areas of 43 streams in the USA^56^. In contrast to these studies, our findings suggest that smooth newt abundance is primarily influenced by the availability of potential breeding sites and the effects of nearby croplands, rather than the proportion of urban land use in the surrounding area.

Additionally, as predicted, we found that the proximity of linear infrastructures, such as roads and railways, negatively impacted smooth newt populations (Table 5; Fig. 2c and d). The negative impact of roads on amphibian population dynamics is well established in urban ecology^57^. Roads and traffic have direct negative effects on amphibians, such as injuries and road-kills^58^, and indirect effects including habitat loss and degradation^16^ increased levels of noise, light^59^ and chemical pollution^60^. For example, a citizen science study found that anuran species richness and individual species distributions were consistently limited by both road density and traffic volume^13^ and it has been shown that roads are a major source of environmental pollutants, with high concentrations found in road mitigation tunnels originating from runoff water^14^. The impact of railways on herpetofauna has not been as widely studied as the effects of roads^61^, even though railways can similarly cause both direct and indirect harm to not only larger animals but also smaller, less mobile species such as reptiles and amphibians^62^. In addition to indirect disturbances such as noise and chemical pollution^63^, railways also act as direct physical barriers. Small-sized animals are often unable to cross them due to their inability to climb the rails, as has been documented for several tortoise species^64–66^. Information on amphibian mortality rates caused by railways is limited and often contradictory. For example, Hamer et al.^32^ observed a significant negative impact of roads, but not railways, on larval amphibian abundance in a study conducted near our research area. Conversely, Budzik and Budzik^40^ reported that railway mortality primarily affected three anuran species - *Bufo bufo*,* Rana temporaria*, and *Pelophylax kl. esculentus* - likely due to dehydration after becoming trapped between railway tracks. Moreover, a study has indicated an unexpectedly high fatality rate among *Rhinella spp.*, primarily due to vehicle collisions and desiccation along the railways of a Brazilian Amazonian railroad, with an estimated 11 toads killed per kilometre annually^67^. In contrast to previous and our findings, Kaczmarski and Kaczmarek^68^ found low mortality rates for smooth newts (*L. vulgaris*), despite their high numbers on the tracks and frequent rail traffic. Our results suggest that, alongside roads, railways also negatively impact smooth newts. Railways act as physical barriers, potentially disrupting metapopulation dynamics, while also introducing indirect stressors, such as pollution and noise, which can affect survival and reproduction. Since many aspects of railway ecology are still under-researched, it is important to gather more empirical data on how railways impact smooth newt populations.

In addition to the landscape-scale covariates identified in our study, local environmental factors are critical in shaping amphibian presence and abundance. Key factors such as hydroperiod^69^, water quality (e.g., transparency, nutrient levels)^70^ and predator presence (invertebrates, fish)^39,71^ are well-known influences on amphibian populations in wetland ecosystems. Among these, aquatic vegetation plays a particularly crucial role in determining habitat quality. Dense vegetation creates microhabitats that support prey diversity, offers refuge from predators and provides structural support for egg deposition, all of which contribute to optimal conditions for both newt larvae and adults^72^. While our study did not include data on these variables, we acknowledge their potential impact on smooth newt populations and suggest that future research explore their effects to gain a deeper understanding of the ecological dynamics within amphibian habitats.

## Conclusions

The spatial scale of land-use effects is a critical factor in shaping how natural and human-modified land use impacts amphibian presence, distribution, and abundance. In our study, we measured these effects at different spatial scales, providing a more comprehensive understanding of their influence on smooth newt populations. Understanding these scales is essential for uncovering the underlying mechanisms, as localized effects are typically associated with direct impacts on individual survival, while larger-scale effects often lead to habitat fragmentation and reduced metapopulation connectivity. Our study revealed that smooth newt abundance is primarily influenced by the accessibility of other potential breeding sites within an intermediate (500 m) proximity to the ponds they occupy, as these nearby sites may enhance metapopulation connectivity for the species. In contrast, we found that cropland coverage plays a significant role in reducing newt abundance at a smaller scale (250 m), suggesting that agricultural activities have more localized effects, typically linked to direct impacts on individual survival, such as reduced availability of terrestrial habitats or pollutant runoff. Urban land use did not seem to have a major impact on abundance, possibly due to this species less strict habitat requirements compared to many other amphibians, enabling them to better tolerate human presence and habitat modifications. Our results offer biologically-based estimates for the protection of terrestrial habitats surrounding wetlands occupied by smooth newts. Our data clearly indicate that a 500-metre buffer zone is an adequate size to ensure a viable habitat for this species, as it is sufficient to maintain metapopulation connectivity while also mitigating the direct effects of nearby agricultural activities.

Additionally, we found that the proximity of linear infrastructures significantly reduces smooth newt abundance in the study area. This reduction is driven by direct effects, such as road mortality, and indirect effects, including traffic noise, altered hydrological patterns, polluted runoff and fragmentation that disrupts metapopulation dynamics. The scale effect of roads (road-effect zone) should be considered in future studies, as research has demonstrated that the impacts of roads on wildlife often extend beyond the immediate vicinity, with significant ecological effects reaching several kilometres in some cases^16^. Similarly, the ‘railway-effect’ zone should also be considered for smooth newts, as our study found that rail tracks significantly reduce their abundance. Railway ecology, especially concerning small amphibians, remains understudied and presents contradictory findings, highlighting the need for further investigation to gain more accurate information about the extent of railway effects on smooth newts.

Given the increasing human population and the corresponding demand for more food supplies and infrastructure development, it is crucial to understand how to conserve suitable aquatic and terrestrial habitats for amphibians in order to mitigate the global decline of these organisms. Conservation management should focus on ensuring adequate accessibility to wetlands for breeding sites and maintaining sufficient distances between sensitive freshwater ecosystems and croplands, roads and railways. Additionally, the introduction of artificial overwintering hibernacula near croplands, as well as migration tunnels and fences for railways and roads, should also be considered as part of effective conservation strategies.

## Methods

### Focal species and study area

The smooth newt (*Lissotriton vulgaris*) is a widely distributed and relatively common semi-aquatic amphibian (Order: Caudata, Family: Salamandridae) found throughout much of Europe^73,74^. While the IUCN Red List considers populations stable, human-induced threats such as habitat fragmentation and isolation significantly affect the species’ population dynamics^75^. This species requires suitable terrestrial habitats during an extended land-dwelling juvenile stage, but can also use lower-quality artificial habitats^38,68^. The newts exhibit strong site fidelity, returning to the same breeding ponds and refuges, but habitat degradation and isolation reduce occurrence and abundance in urban areas^39^. The species prefers calm, shallow ponds with dense vegetation, inhabiting both natural and artificial urban ponds^37,76^. Large lakes with shallow wetland areas can also serve as potential habitats^77^. In Hungary, *L. vulgaris* is widespread, inhabiting areas with suitable waterbodies for reproduction^78^. The country is home to two subspecies: *L. v. ampelensis and L. v. vulgaris*. The latter occurs in western Hungary, including the waterbodies surrounding Lake Balaton^79^.

We conducted our study in waterbodies surrounding Lake Balaton, which is the largest shallow lake in Central Europe, covering 596 km² with an average depth of 3.3 m^77,80,81^. A recent study by Petrovszki et al.^82^ highlights the significant urbanization and human-induced disturbances occurring along the shoreline and surrounding areas of Lake Balaton. The study found substantial changes in land use in the lake’s catchment area between 1855 and 2018. In 1855, the land use was characterized by agricultural areas (38.38%), forests (28.59%), grasslands (14.01%), urban areas (1.58%), water (11.08%), and wetlands (6.36%). By 2018, agricultural areas slightly increased to 39.12%, but there was a significant rise in urban areas (5.97%) and a noticeable decline in wetlands (2.59%). Forests and grasslands also showed notable changes, with forests increasing to 32.85%, while grasslands decreased to 8.44%. These shifts indicate a clear trend of urban and agricultural expansion, while wetland and terrestrial habitats are being increasingly reduced. In addition to urban development along its shoreline, the surrounding area consists largely of agricultural land and is intersected by roads and railways with high traffic density (M7 motorway, road 7 and 71). As a result, the surrounding area of Lake Balaton represents a mixed urban-agricultural landscape extensively fragmented by roads and railways, offering a valuable model system for studying the effects of human-modified environments on *L. vulgaris* and the aquatic ecosystems it inhabits.

Our study area encompassed a 10 km-wide zone of land around Lake Balaton. Initially, we identified 50 potential newt habitats including ponds, floodplain ponds, stormwater ponds, slow-flowing streams, canals and ditches within a 10-km radius around the lake using Google Earth imagery^83^. Thirty-two waterbodies were chosen as final sampling sites based on criteria such as property access, water permanency, land cover characteristics, proximity to roads and railways, and sufficient variability in waterbody size (Fig. 1 and Supplementary Table S1).

### Newt surveys

Two aquatic trap surveys were conducted at each of the 32 sites in 2022 over one breeding season (survey 1: April 4th - April 11th; survey 2: April 13th - April 20th). We sampled in spring to maximize the chances of capturing smooth newts, as the peak of their breeding season occurs from March to April.

The abundance of newts was estimated using Dewsbury traps designed specifically for the safe capture of newts^84^. The number of traps deployed was adjusted according to the waterbody area, with a minimum of 4 traps, plus 2 additional traps for every 0.02 km² of surface area. Streams and waterbodies larger than 1 km² were sampled using 18 traps. Traps were evenly distributed within a 50 × 30 m rectangular area (sampling plot) near the shoreline targeting microhabitats favourable for newts, such as stagnant or slow-flowing sites with dense vegetation^73^. Water depth ranged from 13.83 to 77.03 cm (mean ± SD: 34.14 ± 15.30). Traps were positioned on the bottom of the water column and were deployed in the afternoon and left overnight during the peak activity period of smooth newts^73^. After 24 h, the traps were checked, and any captured newts were counted and released unmarked at the site of capture.

Survey-specific environmental variables were measured and used as detection covariates in the data analysis (Supplementary Table S2). Water temperature (WTEMP) was recorded using Onset HOBO Pendant MX Water Temperature Data Loggers (Onset Computer Corp., Bourne, MA, USA). A data logger was placed in one of the traps during each survey, where it recorded the temperature every 15 min over a 24-hour period. The average of the recorded measurements was used as the WTEMP value for analysis. Water depth (DEPTH) was measured at three random locations within the sampling plots, and the average was used as the value for that survey. The percentage of emergent vegetation (VEG) within the plot was estimated visually by the same person. Julian date (DATE) and the number of traps (TRAPS) were recorded for each survey.

Since the chytrid fungus (*Batrachochytrium dendrobatidis*)^85,86^ and Ranavirus^87^ have already been detected in Hungary, and the presence of *B. salamandrivorans*^88^ is also a possibility, we followed standard hygiene protocols during fieldwork^89^ to minimize the risk of spreading amphibian diseases.

### Site covariates

Environmental variables were assessed within 250, 500, and 1000 m radius of a site (Fig. 1). These buffer zone sizes were selected to include the maximum dispersal range (approximately 500 m) of *L. vulgaris* and other newt species^90^. Landscape-scale land cover covariates were quantified as percentage cover (%) within the three buffer zones (Supplementary Table S2). Urban land cover encompassed artificial surfaces created by human activity, such as buildings, paved roads, dirt roads, railways, urban green spaces, and other developed areas within the three buffer zones (URBAN250, URBAN500, URBAN1000). Cropland/ agricultural land cover encompassed various types of cultivated areas, such as arable lands, fields, vineyards, orchards, berries and other plantations and complex cultivation structures (CROPLAND250, CROPLAND500, CROPLAND1000). Wetland cover included aquatic marsh/swamp vegetation, periodically flooded grasslands, marsh and swamp meadows (WETLAND250, WETLAND500, WETLAND1000). As smooth newts prefer a large variety of non-aquatic habitats^39^ we included grasslands, herbaceous vegetation, forests, and woodlands as natural terrestrial habitats (TERRSITES250, TERRSITES500, TERRSITES1000).

We hypothesized that roads and railways present significant mortality risks for newts^13,32,40^. Based on previous research^32,91,92^, we also assumed that major roads, including 2-lane main roads and 4-lane highways, impose a greater barrier to amphibian movement and a higher risk of road mortality and pollution exposure compared to secondary roads. In our study area, the primary high-traffic roads are Road 7, Road 71 and the M7 motorway which encircle Lake Balaton and experience consistently high traffic throughout the year. Consequently, variables related to road impacts were assessed extending from each site: (1) distance (km) to the nearest major 2- and 4-lane roads (M7 motorway, road 7 and 71) (DISTMAINROAD); and (2) distance to the nearest parallel railway lines (DISTRAILWAY).

Larger habitat patches can support larger animal population sizes^93^ hence, waterbody area (WATERBODY) was measured for all sites. Moreover, the nearest neighbour distances (NNDIST) of the sampling sites were calculated to evaluate spatial autocorrelation (see Statistical analyses). The summary of the mean, SD, minimum, and maximum values of all covariates is presented in a Table of the supplementary material (Supplementary Table S2).

We used Google Earth Pro, QGIS version 3.28.0^94^ and The Ecosystem Map of Hungary^95^ for all landscape and distance measurements.

### Statistical analyses

Hierarchical N-mixture models were used within a Bayesian framework to analyse newt survey count data^96,97^. These models estimate abundance based on repeated counts of a species at a site while accounting for imperfect detection. Previous studies have shown that N-mixture models provide reliable abundance estimates comparable to capture-mark-recapture methods^98,99^. The model assumes abundance (N_i_) at site i follows a Poisson distribution with expected abundance (λ) across sites and uses repeated counts (C_ij_) at each site during surveys to estimate detection probability (p), with counts following a Binomial distribution (N_i_, p)^96,97^. Environmental covariates can be incorporated to express abundance and detection as functions of these factors, using log or logit links. As ectothermic animals, the detectability of amphibians is largely influenced by environmental temperature^93^. Habitat structure (e.g. vegetation) and waterbody depth may also affect the probability of detecting aquatic amphibians^100^. Consequently, we assumed that smooth newts would be more detectable in warmer water, in areas with greater vegetation, and in shallower waterbodies. Additionally, because detectability often increases later in the mating season, we assumed higher detectability on later spring dates and expected greater sampling effort to positively impact newt detection. Based on these assumptions, we included WTEMP, VEG, DEPTH, DATE, and TRAPS as variables potentially influencing the detection of smooth newts. Consequently, five models were developed to identify which detection covariate(s) significantly influenced the detectability of smooth newts. The detection sub-model assumed that detection followed a Binomial distribution and the detection probability was modeled as a logit-linear function of a single survey-specific covariate:

logit (p_ij_) = α_0_ + α_1_ × Y_ij_.

where α_0_ is the intercept, α_1_ denotes the effect of the covariate, and Y corresponds to one of the detection covariates for each survey^97^. The five detection models were evaluated using the Watanabe-Akaike Information Criterion (WAIC)^101,102^ to identify the most supported detection covariate. Models with a ΔWAIC < 2 were regarded as having the strongest support^103^.

The abundance sub-model assumed that the abundance at a site follows a Poisson distribution and the mean abundance was expressed as a log-linear function of the site covariates:

$${\text{log }}({{\text{l}}_{\text{i}}}){\text{ }}={{\text{b}}_0}\,+\,{{\text{b}}_{\text{1}}}\, \times \,{{\text{X}}_{\text{i}}}\,+\,{{\text{b}}_{\text{2}}}\, \times \,{{\text{X}}_{\text{i}}}\,+\,{{\text{b}}_{\text{3}}}\, \times \,{{\text{X}}_{\text{i}}}\,+\,{{\text{e}}_{\text{i}}}$$

where β_0_ is the intercept, β_1_, β_2_ and β_3_ are covariate effects and X is one of the landscape or local covariates at site i (Supplementary Table S2)^97^. To address overdispersion and unexplained variation in abundance across sites, we incorporated a random effect (ε) in each model^104,105^. This accounted for potential random influences from unmeasured environmental factors and variations, such as local-scale within-pond features (e.g. fish presence/absence, aquatic vegetation, water chemistry).To determine the most appropriate spatial scale to test our predictions, we constructed three models for each land use covariate across the three different scales (250, 500, 1000 m), resulting in a total of twelve models. The best-supported models were selected based on the ΔWAIC values. In the final model set, we assessed the influence of various covariates on the abundance of smooth newts by constructing four models, each including a site covariate (URBAN, CROPLAND, WETLAND, or TERRSITES) on the most supported scale. To evaluate the impact of linear structures such as roads and railways, we also developed two additional models incorporating DISTROAD and DISTRAILWAY. The best supported models from the final six models were also selected based on their ΔWAIC values. Since larger waterbodies can support more abundant newt populations^106^, the area of the sampled waterbody (WATERBODY) was included as an offset variable in all models. Additionally, the nearest neighbour distance (NNDIST) was incorporated in all models, which has been consistently proven to effectively address spatial autocorrelation among sites^107^ (Supplementary Table S3). Following the recommendation to maintain a minimum ratio of ten sites per estimated parameter (32/3 = 10.7), we limited each sub-model to a maximum of three covariates^108^, ensuring that each sub-model included only one site covariate in addition to WATERBODY and NNDIST. Prior to building the models, we standardized all covariates (mean = 0; standard deviation = 1) to improve model convergence, and assessed each for collinearity, applying a cut-off for inclusion^109^ based on a Pearson correlation coefficient (R) < 0.7. None of the site covariates showed strong correlation (*R* < 0.62). Additionally, WATERBODY, NNDIST, DISTROAD and DISTRAILWAY were log-transformed before standardization.

In the models, we ran three replicates of Markov Chain Monte Carlo (MCMC), generating a total of 650,000 samples from the posterior distribution, with an initial “burn-in” phase of 50,000 samples and a thinning rate of 13. Model convergence was considered satisfactory if the Brooks-Gelman-Rubin statistic (R̂)^110^ was less than 1.1 and through visual inspection of traceplots. For the best-supported models, we present the mean, standard deviation (SD) and 95% Bayesian credible intervals (BCI) for all parameters. Parameter estimates were deemed important when the BCI did not overlap zero, although a small degree of overlap with zero was tolerated^111^. We also reported the proportion of the posterior distribution (*f*), which can be interpreted as the probability that the effect is either positive or negative^112,113^. Covariate relationships were also considered important if *f* > 0.90. All modelling was conducted using JAGS version 4.3.0^114^, accessed through the R2jags package^115^ in R^116^.

## Electronic supplementary material

Below is the link to the electronic supplementary material.

Supplementary Material 1

## Data Availability

The datasets that support the findings of this study are available on request to the corresponding author.

## References

[1] Ellis, E. C. et al. People have shaped most of terrestrial nature for at least 12,000 years. *P Natl. Acad. Sci. USA*. **118**10.1073/pnas.2023483118 (2021). 10.1073/pnas.2023483118 PMC8092386 33875599

[2] Fardila, D., Kelly, L. T., Moore, J. L. & McCarthy, M. A. A systematic review reveals changes in where and how we have studied habitat loss and fragmentation over 20 years. *Biol. Conserv.* **212**, 130–138. 10.1016/j.biocon.2017.04.031 (2017).

[3] Haddad, N. M. et al. Habitat fragmentation and its lasting impact on Earth’s ecosystems. *Sci. Adv.* **1**10.1126/sciadv.1500052 (2015). 26601154 10.1126/sciadv.1500052 PMC4643828

[4] Sayer, C. A. et al. One-quarter of freshwater fauna threatened with extinction. *Nature* **638**, 138–145. 10.1038/s41586-024-08375-z (2025). 39779863 10.1038/s41586-024-08375-z PMC11798842

[5] Cerda-Peña, C. & Rau, J. R. The importance of wetland habitat area for waterbird species-richness. *Ibis* **165**, 739–752. 10.1111/ibi.13205 (2023).

[6] Gibbs, J. P. Importance of small wetlands for the persistence of local-populations of wetland-associated animals. *Wetlands* **13**, 25–31. 10.1007/bf03160862 (1993).

[7] Kingsford, R. T., Basset, A. & Jackson, L. Wetlands: conservation’s poor cousins. *Aquat. Conserv.* **26**, 892–916. 10.1002/aqc.2709 (2016).

[8] Hamer, A. J. & McDonnell, M. J. Amphibian ecology and conservation in the urbanising world: A review. *Biol. Conserv.* **141**, 2432–2449. 10.1016/j.biocon.2008.07.020 (2008).

[9] Luedtke, J. A. et al. Ongoing declines for the world’s amphibians in the face of emerging threats. *Nature* **622**, 308. 10.1038/s41586-023-06578-4 (2023). 37794184 10.1038/s41586-023-06578-4 PMC10567568

[10] Hulley, M. The urban heat Island effect: causes and potential solutions. in Woodhead Publishing Series in Energy, Metropolitan Sustainability (ed (ed Zeman, F.) 79–98 (Woodhead Publishing, (2012).

[11] Grimm, N. B. et al. Global change and the ecology of cities. *Science* **319**, 756–760. 10.1126/science.1150195 (2008). 18258902 10.1126/science.1150195

[12] Elmqvist, T. et al. Urbanization, Biodiversity and Ecosystem Services: Challenges and Opportunities: A Global AssessmentSpringer Netherlands,. (2013).

[13] Cosentino, B. J. et al. Citizen science reveals widespread negative effects of roads on amphibian distributions. *Biol. Conserv.* **180**, 31–38. 10.1016/j.biocon.2014.09.027 (2014).

[14] White, K. J., Petrovan, S. O. & Mayes, W. M. Pollutant accumulation in road mitigation tunnels for amphibians: A multisite comparison on an ignored but important issue. *Front. Ecol. Evol.* **11**, 10. 10.3389/fevo.2023.1133253 (2023).

[15] Goessens, T. et al. Agricultural contaminants in amphibian breeding ponds: occurrence, risk and correlation with agricultural land use. *Sci. Total Environ.* **806**10.1016/j.scitotenv.2021.150661 (2022). 10.1016/j.scitotenv.2021.150661 34597541

[16] Dixon, H. J. et al. The effects of roadways on lakes and ponds: A systematic review and assessment of knowledge gaps. *Environ. Rev.* **23**10.1139/er-2022-0022 (2022).

[17] Fahrig, L. et al. Is habitat fragmentation bad for biodiversity? *Biol. Conserv.* **230**, 179–186. 10.1016/j.biocon.2018.12.026 (2019).

[18] Fletcher, R. J. et al. Is habitat fragmentation good for biodiversity? *Biol. Conserv.* **226**, 9–15. 10.1016/j.biocon.2018.07.022 (2018).

[19] Cayuela, H., Lambrey, J., Vacher, J. P. & Miaud, C. Highlighting the effects of land-use change on a threatened amphibian in a human-dominated landscape. *Popul. Ecol.* **57**, 433–443. 10.1007/s10144-015-0483-4 (2015).

[20] Ribeiro, P. V. A. et al. Effects of urbanisation and pollution on the heterophil/lymphocyte ratio in birds from Brazilian Cerrado. *Environ. Sci. Pollut Res.* **29**, 40204–40212. 10.1007/s11356-022-19037-w (2022). 10.1007/s11356-022-19037-w 35119632

[21] van Vliet, J. Direct and indirect loss of natural area from urban expansion. *Nat. Sustain.* **2**, 755–763. 10.1038/s41893-019-0340-0 (2019).

[22] Zhang, W. et al. Responses of Anuran communities to rapid urban growth in Shanghai, China. *Urban Urban Gree*. **20**, 365–374. 10.1016/j.ufug.2016.10.005 (2016).

[23] Bodo, T., Gimah, B. G. & Seomoni, K. J. Deforestation and habitat loss: Human causes, consequences and possible solutions. *J. Geographical Res.* **4**, 22–30 (2021).

[24] Curado, N., Hartel, T. & Arntzen, J. W. Amphibian pond loss as a function of landscape change - A case study over three decades in an agricultural area of Northern France. *Biol. Conserv.* **144**, 1610–1618. 10.1016/j.biocon.2011.02.011 (2011).

[25] Rytwinski, T. & Fahrig, L. Do species life history traits explain population responses to roads? A meta-analysis. *Biol. Conserv.* **147**, 87–98. 10.1016/j.biocon.2011.11.023 (2012).

[26] Scheffers, B. R. & Paszkowski, C. A. The effects of urbanization on North American amphibian species: identifying new directions for urban conservation. *Urban Ecosyst.* **15**, 133–147. 10.1007/s11252-011-0199-y (2012).

[27] Clauzel, C. et al. From single to multiple habitat connectivity: The key role of composite ecological networks for amphibian conservation and habitat restoration. *Biol. Conserv.* **289**, 12. 10.1016/j.biocon.2023.110418 (2024).

[28] Marsh, D. M. et al. Effects of roads and land use on frog distributions across Spatial scales and regions in the Eastern and central united States. *Divers. Distrib.* **23**, 158–170. 10.1111/ddi.12516 (2017).

[29] Campos, F. S. et al. Searching for networks: Ecological connectivity for amphibians under climate change. *Environ. Manage.* **65**, 46–61. 10.1007/s00267-019-01240-0 (2020). 31832730 10.1007/s00267-019-01240-0

[30] Semlitsch, R. D. Critical elements for biologically based recovery plans of aquatic-breeding amphibians. *Conserv. Biol.* **16**, 619–629. 10.1046/j.1523-1739.2002.00512.x (2002).

[31] Semlitsch, R. D. & Bodie, J. R. Biological criteria for buffer zones around wetlands and riparian habitats for amphibians and reptiles. *Conserv. Biol.* **17**, 1219–1228. 10.1046/j.1523-1739.2003.02177.x (2003).

[32] Hamer, A. J., Barta, B., Bohus, A., Gal, B. & Schmera, D. Roads reduce amphibian abundance in ponds across a fragmented landscape. *Glob Ecol. Conserv.* **28**10.1016/j.gecco.2021.e01663 (2021).

[33] Houlahan, J. E. & Findlay, C. S. Estimating the ‘critical’ distance at which adjacent land-use degrades wetland water and sediment quality. *Landsc. Ecol.* **19**, 677–690. 10.1023/b:Land.0000042912.87067.35 (2004).

[34] Pellet, J., Hoehn, S. & Perrin, N. Multiscale determinants of tree frog (*Hyla arborea*) calling ponds in Western Switzerland. *Biodivers. Conserv.* **13**, 2227–2235. 10.1023/B:BIOC.0000047904.75245.1f (2004).

[35] Covarrubias, S., González, C. & Gutiérrez-Rodríguez, C. Effects of natural and anthropogenic features on functional connectivity of anurans: a review of landscape genetics studies in temperate, subtropical and tropical species. *J. Zool.* **313**, 159–171. 10.1111/jzo.12851 (2021).

[36] Sawatzky, M. E., Martin, A. E. & Fahrig, L. Landscape context is more important than wetland buffers for farmland amphibians. *Agr Ecosyst. Environ.* **269**, 97–106. 10.1016/j.agee.2018.09.021 (2019).

[37] Rannap, R. et al. Constructed wetlands as potential breeding sites for amphibians in agricultural landscapes: A case study. *Ecol. Eng.* **158**, 9. 10.1016/j.ecoleng.2020.106077 (2020).

[38] Heiss, E., Aerts, P. & Van Wassenbergh, S. Flexibility is everything: Prey capture throughout the seasonal habitat switches in the smooth Newt *Lissotriton vulgaris*. *Org. Divers. Evol.* **15**, 127–142. 10.1007/s13127-014-0187-1 (2015). 26097413 10.1007/s13127-014-0187-1 PMC4470538

[39] Vehkaoja, M., Thompson, S. M. A., Niemi, M. & Väänänen, V. M. Connectivity, land use, and fish presence influence smooth Newt (*Lissotriton vulgaris*) occurrence and abundance in an urban landscape. *Front. Ecol. Evol.* **11**, 12. 10.3389/fevo.2023.1157297 (2023).

[40] Budzik, K. A. & Budzik, K. M. A preliminary report of amphibian mortality patterns on railways. *Acta Herpetol*. **9**, 103–107 (2014).

[41] Falaschi, M. et al. Explaining declines of Newt abundance in Northern Italy. *Freshw. Biol.* **67**, 1174–1187. 10.1111/fwb.13909 (2022).

[42] Denoël, M., Perez, A., Cornet, Y. & Ficetola, G. F. Similar local and landscape processes affect both a common and a rare Newt species. *Plos One*. **8**10.1371/journal.pone.0062727 (2013). 10.1371/journal.pone.0062727 PMC3643927 23658765

[43] Revilla, E. & Wiegand, T. Individual movement behavior, matrix heterogeneity, and the dynamics of spatially structured populations. *P Natl. Acad. Sci. USA*. **105**, 19120–19125. 10.1073/pnas.0801725105 (2008). 10.1073/pnas.0801725105 PMC2614725 19060193

[44] Joly, P., Miaud, C., Lehmann, A. & Grolet, O. Habitat matrix effects on pond occupancy in newts. *Conserv. Biol.* **15**, 239–248. 10.1046/j.1523-1739.2001.99200.x (2001).

[45] Manenti, R., Falaschi, M., Delle Monache, D., Marta, S. & Ficetola, G. F. Network-scale effects of invasive species on spatially-structured amphibian populations. *Ecography* **43**, 119–127. 10.1111/ecog.04571 (2020).

[46] Dalpasso, A. et al. Similar species, different fates: Abundance dynamics in spatially structured populations of common and threatened frogs. *Divers. Distrib.* **28**, 770–781. 10.1111/ddi.13483 (2022).

[47] Hodgson, J. A., Thomas, C. D., Wintle, B. A. & Moilanen, A. Climate change, connectivity and conservation decision making: Back to basics. *J. Appl. Ecol.* **46**, 964–969. 10.1111/j.1365-2664.2009.01695.x (2009).

[48] Suárez, R. P. et al. Anuran responses to spatial patterns of agricultural landscapes in Argentina. *Landsc. Ecol.* **31**, 2485–2505. 10.1007/s10980-016-0426-2 (2016).

[49] Carvalho, F. P. Pesticides, environment, and food safety. *Food Energy Secur.* **6**, 48–60 (2017).

[50] Mann, R. M., Hyne, R. V., Choung, C. B. & Wilson, S. P. Amphibians and agricultural chemicals: Review of the risks in a complex environment. *Environ. Pollut.* **157**, 2903–2927. 10.1016/j.envpol.2009.05.015 (2009). 19500891 10.1016/j.envpol.2009.05.015

[51] Davidson, C. & Knapp, R. A. Multiple stressors and amphibian declines: Dual impacts of pesticides and fish on yellow-legged frogs. *Ecol. Appl.* **17**, 587–597. 10.1890/06-0181 (2007). 17489262 10.1890/06-0181

[52] Johnson, P. T. J. et al. Aquatic eutrophication promotes pathogenic infection in amphibians. *P Natl. Acad. Sci. USA*. **104**, 15781–15786. 10.1073/pnas.0707763104 (2007). 10.1073/pnas.0707763104 PMC2000446 17893332

[53] Joly, P. Behavior in a changing landscape: Using movement ecology to inform the conservation of Pond-Breeding amphibians. *Front. Ecol. Evol.* **7**, 17. 10.3389/fevo.2019.00155 (2019).

[54] Green, J., Govindarajulu, P. & Higgs, E. Multiscale determinants of Pacific chorus frog occurrence in a developed landscape. *Urban Ecosyst.* **24**, 587–600. 10.1007/s11252-020-01057-4 (2021). 34720571 10.1007/s11252-020-01057-4 PMC8550069

[55] Woodford, J. E. & Meyer, M. W. Impact of lakeshore development on green frog abundance. *Biol. Conserv.* **110**, 277–284. 10.1016/s0006-3207(02)00230-6 (2003).

[56] Hoffmann, A. A. & Sgrò, C. M. Climate change and evolutionary adaptation. *Nature* **470**, 479–485. 10.1038/nature09670 (2011). 21350480 10.1038/nature09670

[57] Trombulak, S. C. & Frissell, C. A. Review of ecological effects of roads on terrestrial and aquatic communities. *Conserv. Biol.* **14**, 18–30. 10.1046/j.1523-1739.2000.99084.x (2000).

[58] Andelkovic, M. & Bogdanovic, N. Amphibian and reptile road mortality in special nature reserve Obedska Bara, Serbia. *Animals* **12** (15). 10.3390/ani12050561 (2022). 35268129 10.3390/ani12050561 PMC8908848

[59] Cronin, A. D., Smit, J. A. & Halfwerk, W. Anthropogenic noise and light alter Temporal but not Spatial breeding behavior in a wild frog. *Behav. Ecol.* **33**, 1115–1122. 10.1093/beheco/arac077 (2022). 36518635 10.1093/beheco/arac077 PMC9735234

[60] Forgione, M. E. & Brady, S. P. Road salt is more toxic to wood frog embryos from polluted ponds. *Environ. Pollut.* **296**, 118757. 10.1016/j.envpol.2021.118757 (2022). 34973378 10.1016/j.envpol.2021.118757

[61] Borda-de-Água, L., Barrientos, R., Beja, P. & Pereira, H. M. *Railway Ecology* (Springer Nature, 2017).

[62] Barrientos, R., Ascensao, F., Beja, P., Pereira, H. M., & Borda-de-Agua, L. Railway ecology vs. road ecology: Similarities and differences. *Eur. J. Wildl. Res.* **65**, 9. 10.1007/s10344-018-1248-0 (2019).

[63] Lucas, P. S., de Carvalho, R. G. & Grilo, C. Railway disturbances on wildlife: types, effects, and mitigation measures. in Railway Ecology (eds (eds Borda-de-Água, L., Barrientos, R., Beja, P. & Pereira, H.) 81–99 (Springer, (2017).

[64] Iosif, R. Railroad-associated mortality hot spots for a population of Romanian Hermann’s tortoise (T*estudo hermanni boettgeri*): a gravity model for railroad-segment analysis. *Procedia Environ. Sci.* **14**, 123–131. 10.1016/j.proenv.2012.03.012 (2012).

[65] Kornilev, Y. V., Price, S. J. & Dorcas, M. E. Between a rock and hard place: Responses of Eastern box turtles (*Terrapene carolina*) when trapped between railroad tracks. *Herpetological Rev.* **37**, 145–148 (2006).

[66] Rautsaw, R. M. et al. Stopped dead in their tracks: The impact of railways on gopher tortoise (*Gopherus polyphemus*) movement and behavior. *Copeia* **106**, 135–143. 10.1643/CE-17-635 (2018).

[67] Dornas, R. A. P., Teixeira, F. Z., Gonsioroski, G. & Nóbrega, R. A. A. Strain by the train: patterns of Toad fatalities on a Brazilian Amazonian railroad. *Sci. Total Environ.* **660**, 493–500. 10.1016/j.scitotenv.2018.12.371 (2019). 30640116 10.1016/j.scitotenv.2018.12.371

[68] Kaczmarski, M. & Kaczmarek, J. M. Heavy traffic, low mortality - tram tracks as terrestrial habitat of newts. *Acta Herpetol*. **11**, 227–231. 10.13128/Acta_Herpetol-17922 (2016).

[69] Hamer, A. J. et al. Hydrology is a major influence on amphibian abundance in a large European floodplain. *Freshw. Biol.* **68**, 1303–1318. 10.1111/fwb.14104 (2023). 38516301 10.1111/fwb.14104 PMC10952816

[70] Cirovic, R., Radovic, D. & Vukov, T. D. Breeding site traits of European newts (*Triturus macedonicus, Lissotriton vulgaris, and Mesotriton alpestris*: Salamandridae) in the Montenegrin karst region. *Arch. Biol. Sci.* **60**, 459–468. 10.2298/abs0803459c (2008).

[71] Cai, W. et al. Assembly processes inferred from eDNA surveys of a pond metacommunity are consistent with known species ecologies. *Ecography* **15**10.1111/ecog.07461 (2025).

[72] Burrow, A. & Maerz, J. How plants affect amphibian populations. *Biol. Rev.* **97**, 1749–1767. 10.1111/brv.12861 (2022). 35441800 10.1111/brv.12861

[73] AmphibiaWeb. Lissotriton vulgaris,. (2025).

[74] IUCN Red List. *Smooth newt (Lissotriton vulgaris)*, (2025). https://www.iucnredlist.org/

[75] Denöel, M. & Ficetola, G. F. Landscape-level thresholds and newt conservation. *Ecol Appl* 17, 302–309, (2007). 10.1890/1051-0761017[0302:Ltanc]2.0.Co;2 (2007). 10.1890/1051-0761(2007)017[0302:ltanc]2.0.co;2 17479853

[76] Skei, J. K., Dolmen, D., Ronning, L. & Ringsby, T. H. Habitat use during the aquatic phase of the newts *Triturus vulgaris* (L.) and *T. cristatus* (Laurenti) in central Norway: proposition for a conservation and monitoring area. *Amphibia-Reptilia* **27**, 309–324. 10.1163/156853806778189972 (2006).

[77] Tóth, S. *A Bakony-vidék és a Balaton-medence herpetofaunája (Amphibia–Reptilia)*. Vol. 34 (Magyar Természettudományi Múzeum, (2015).

[78] Országos Kétéltű- és Hüllőtérképezés. *Pettyes gőte (Lissotriton vulgaris)*, (2025). https://herpterkep.mme.hu/keteltu.php?lang=hu&id=14

[79] Herczeg, D. et al. Genomic analysis reveals complex population structure within the smooth Newt, *Lissotriton vulgaris*, in central Europe. *Ecol. Evol.* **13**, 12. 10.1002/ece3.10478 (2023). 10.1002/ece3.10478 PMC10469019 37664508

[80] Istvánovics, V. et al. Updating water quality targets for shallow lake Balaton (Hungary), recovering from eutrophication. *Hydrobiologia* **581**, 305–318. 10.1007/s10750-006-0509-1 (2007).

[81] Specziár, A. & Bíró, P. Spatial distribution and short-term changes of benthic macrofauna in lake Balaton (Hungary). *Hydrobiologia* **389**, 203–216 (1998).

[82] Petrovszki, J., Szilassi, P. & Eros, T. Mass tourism generated urban land expansion in the catchment of lake Balaton, Hungary - analysis of long-term changes in characteristic socio-political periods. *Land. Use Pol.* **142**, 12. 10.1016/j.landusepol.2024.107185 (2024).

[83] Google Inc. *Google Earth*, < (2020). https://earth.google.com/

[84] Dewsbury, D. A novel, effective and safe Newt trap. *Wasserfallen für Amphibien–praktische Anwendung Im Artenmonitoring.–Abhandlungen Aus dem westfälischen*. *Museum Für Naturkunde*. **77**, 189–208 (2014).

[85] Vörös, J. et al. Batrachochytrium dendrobatidis in Hungary: an overview of recent and historical occurrence. *Acta Herpetol*. **13**, 125–140. 10.13128/Acta_Herpetol-22611 (2018).

[86] Harmos, K. et al. Amphibian mortality associated with chytridiomycosis in Central-Eastern Europe. *Herpetology Notes*. **14**, 1213–1218 (2021).

[87] Vörös, J., Herczeg, D., Papp, T., Monsalve-Carcaño, C. & Bosch, J. First detection of ranavirus infection in amphibians in Hungary. *Herpetology Notes*. **13**, 213–217 (2020).

[88] Kostanjsek, R., Turk, M., Vek, M., Gutierrez-Aguirre, I. & Cimerman, N. G. First screening for batrachochytrium dendrobatidis, B. salamandrivorans and ranavirus infections in wild and captive amphibians in Slovenia. *Salamandra* **57**, 162–166 (2021).

[89] Phillott, A. D. et al. Minimising exposure of amphibians to pathogens during field studies. *Dis. Aquat. Organ.* **92**, 175–185. 10.3354/dao02162 (2010). 21268979 10.3354/dao02162

[90] Kovar, R., Brabec, M., Vita, R. & Bocek, R. Spring migration distances of some central European amphibian species. *Amphibia-Reptilia* **30**, 367–378. 10.1163/156853809788795236 (2009).

[91] Eigenbrod, F., Hecnar, S. J. & Fahrig, L. Accessible habitat: an improved measure of the effects of habitat loss and roads on wildlife populations. *Landsc. Ecol.* **23**, 159–168. 10.1007/s10980-007-9174-7 (2008).

[92] Fahrig, L., Pedlar, J. H., Pope, S. E., Taylor, P. D. & Wegner, J. F. Effect of road traffic on amphibian density. *Biol. Conserv.* **73**, 177–182. 10.1016/0006-3207(94)00102-v (1995).

[93] Cooke, A. & Frazer, J. Characteristics of Newt breeding sites. *J. Zool.* **178**, 223–236 (1976).

[94] QGIS 3.28.4-Firenze. QGIS Geographic Information System. Open Source Geospatial Foundation Project. (2022). http://qgis.org

[95] Ministry of Agriculture. in. *The Ecosystem Map of Hungary* (Ministry of Agriculture, Hungary, 2019).

[96] Royle, J. A. N-mixture models for estimating population size from spatially replicated counts. *Biometrics* **60**, 108–115. 10.1111/j.0006-341X.2004.00142.x (2004). 15032780 10.1111/j.0006-341X.2004.00142.x

[97] Royle, J. A., Nichols, J. D. & Kéry, M. Modelling occurrence and abundance of species when detection is imperfect. *Oikos* **110**, 353–359. 10.1111/j.0030-1299.2005.13534.x (2005).

[98] Ficetola, G. F. et al. N-mixture models reliably estimate the abundance of small vertebrates. *Sci. Rep-Uk*. **8**, 8. 10.1038/s41598-018-28432-8 (2018). 10.1038/s41598-018-28432-8 PMC6037707 29985399

[99] Neubauer, G., Wolska, A., Rowinski, P. & Wesolowski, T. N-mixture models estimate abundance reliably: A field test on marsh tit using time-for-space substitution. *Ornithol. Appl.* **124**, 13. 10.1093/ornithapp/duab054 (2022).

[100] Curtis, A. E. & Paton, P. W. C. Assessing detection probabilities of larval amphibians and macroinvertebrates in isolated ponds. *Wetlands* **30**, 901–914. 10.1007/s13157-010-0088-9 (2010).

[101] Watanabe, S. Asymptotic equivalence of Bayes cross validation and widely applicable information criterion in singular learning theory. *J. Mach. Learn. Res.* **11**, 3571–3594 (2010).

[102] Gelman, A., Hwang, J. & Vehtari, A. Understanding predictive information criteria for bayesian models. *Stat. Comput.* **24**, 997–1016. 10.1007/s11222-013-9416-2 (2014).

[103] McCarthy, M. *Bayesian Methods for Ecology* (Cambridge University, 2007).

[104] Kéry, M. et al. Trend Estimation in populations with imperfect detection. *J. Appl. Ecol.* **46**, 1163–1172. 10.1111/j.1365-2664.2009.01724.x (2009).

[105] Knape, J. et al. Sensitivity of binomial N-mixture models to overdispersion: the importance of assessing model fit. *Methods Ecol. Evol.* **9**, 2102–2114. 10.1111/2041-210x.13062 (2018).

[106] Babbitt, K. J. The relative importance of wetland size and hydroperiod for amphibians in Southern new Hampshire, USA. *Wetl Ecol. Manag*. **13**, 269–279. 10.1007/s11273-004-7521-x (2005).

[107] Prugh, L. R. An evaluation of patch connectivity measures. *Ecol. Appl.* **19**, 1300–1310. 10.1890/08-1524.1 (2009). 19688936 10.1890/08-1524.1

[108] Harrison, X. A. et al. A brief introduction to mixed effects modelling and multi-model inference in ecology. *Peerj* **6**, 32. 10.7717/peerj.4794 (2018). 10.7717/peerj.4794 PMC5970551 29844961

[109] Dormann, C. F. et al. Collinearity: a review of methods to deal with it and a simulation study evaluating their performance. *Ecography* **36**, 27–46. 10.1111/j.1600-0587.2012.07348.x (2013).

[110] Brooks, S. P. & Gelman, A. General methods for monitoring convergence of iterative simulations. *J. Comput. Graph Stat.* **7**, 434–455. 10.2307/1390675 (1998).

[111] Cumming, G. & Finch, S. Inference by eye - Confidence intervals and how to read pictures of data. *Am. Psychol.* **60**, 170–180. 10.1037/0003-066x.60.2.170 (2005). 15740449 10.1037/0003-066X.60.2.170

[112] Schmidt, B. R., Brenneisen, S. & Zumbach, S. Evidence-Based amphibian conservation: A case study on Toad tunnels. *Herpetologica* **76**, 228–239. 10.1655/0018-0831-76.2.228 (2020).

[113] Wade, P. R. Bayesian methods in conservation biology. *Conserv. Biol.* **14**, 1308–1316. 10.1046/j.1523-1739.2000.99415.x (2000).

[114] Version, J. A. G. S. 4.3.0 User manual (2017).

[115] Package ’R2jags’. Using R to Run’JAGS’ V. 05–7 (2015).

[116] R: A language and environment for statistical computing. R Foundation for Statistical Computing, Vienna, Austria, (2021).

