# Supplementary material for: Scale-dependent landscape variables and linear infrastructures influence smooth newt (Lissotriton vulgaris) abundance in wetlands of a heavily urbanized lake
Source: Sci Rep. 2025 Apr 22;15:13882. doi: 10.1038/s41598-025-97988-z (PMC12015229; doi:10.1038/s41598-025-97988-z)
Supplement: Supplementary file 1 — Supplementary Material 1 [file 41598_2025_97988_MOESM1_ESM.pdf]

Article title: Scale-dependent landscape variables and linear infrastructures influence smooth newt (*Lissotriton vulgaris*) abundance in wetlands of a heavily urbanized lake

Authors

Boglárka Mészáros (ORCID: 0000-0003-0005-8678) [1, 2 *]
József Bürgés (ORCID: 0009-0008-2247-1870) [1, 3]
Mónika Tamás [1]
Blanka Gál (ORCID: 0000-0001-8513-3010) [1]
Judit Vörös (ORCID: 0000-0001-9707-1443) [1]
Andrew J. Hamer (ORCID: 0000-0001-6031-7841) [4, 5, †]
Dénes Schmera (ORCID: 0000-0003-1248-8413) [1, 2 †]

Affiliations:

[1] HUN-REN Balaton Limnological Research Institute, Klebelsberg Kuno street 3., H-8237 Tihany, Hungary
[2] National Multidisciplinary Laboratory for Climate Change, HUN-REN Balaton Limnological Research Institute, Klebelsberg Kuno street 3, H-8237 Tihany, Hungary
[3] Limnology Research Group, Center of Natural Sciences, University of Pannonia, Egyetem u. 10, Veszprém 8200, Hungary
[4] Institute of Aquatic Ecology, HUN-REN Centre for Ecological Research, Karolina út. 29, 1113 Budapest, Hungary
[5] National Multidisciplinary Laboratory for Climate Change, HUN-REN Centre for Ecological Research, Karolina út. 29, 1113 Budapest, Hungary

† Contributed equally
*Corresponding author

Corresponding author

Boglárka Mészáros
e-mail: meszaros.boglarka@blki.hun-ren.hu
telephone: +36 87 448 244/221

Table S1 ID, name, WGS geographic coordinates, and the number of smooth newts captured at each survey of the 32 locations.

ID	Name	Type	Latitude, N	Longitude, E	Survey 1	Survey 2
1	Köcsi-tó	pond	47.006328282636	17.9972068539821	0	0
2	Badacsonytördemic	marsh	46.8039778250451	17.4681517757251	0	0
3	Keszthely	pond	46.7660118907781	17.2605182060223	0	0
4	Vászoly	pond	46.9431166666667	17.758	0	0
5	Fonyód-U27	pond	46.7234141776905	17.5408122605298	12	3
6	Fonyód-U28	pond	46.7130820923472	17.5348384806479	26	16
7	Balatonszemes-U37	stormwater pond	46.78963530758	17.7468052788598	6	3
8	Balatonőszöd-1	pond	46.8102708431951	17.8080422046144	0	0
9	Balatonőszöd-2	pond	46.8124666666667	17.80825	1	1
10	Balatonőszöd-3	pond	46.8112485251791	17.8080155946086	3	0
11	Balatonszemes-2	canal	46.8043166666667	17.7756166666667	0	1
12	Balatonszemes-1	pond	46.7982674276347	17.7461558630548	2	2
13	Balatonlelle	canal	46.7904666666667	17.7289666666667	3	4
14	Bugaszegi-tó	pond	46.7509549793134	17.6618645115592	6	6
15	Jamai-patak	slow-flowing stream	46.7504833333333	17.6581666666667	42	26
16	Szentgyörgyi-árok	canal	46.7677	17.6355333333333	8	10
17	Ordacsehi-berek	marsh	46.7515194661595	17.6016651460074	8	3
18	Fonyód	canal	46.7332333333333	17.5621	7	3
19	Balatonfenyves-1	pond	46.7130666666667	17.5029833333333	1	1
20	Balatonfenyves-2	pond	46.7099538868683	17.4961509855538	1	1
21	Balatonkeresztúr	pond	46.6964833333333	17.3917333333333	6	0
22	Balatonederics	marsh	46.8150852524874	17.3872141477153	1	1
23	Eger-víz berke	floodplain pond	46.8067915723506	17.4598665548209	2	0
24	Badacsonytomaj	marsh	46.8082620053976	17.5188029952667	6	0
25	Salföld	pond	46.8355833333333	17.5525166666667	14	19
26	Kornyi-tó	pond	46.8617166666667	17.5917	0	4
27	Aszófői-séd	slow-flowing stream	46.9260291813467	17.8440100392634	1	0
28	Külső-tó	pond	46.9129147321446	17.8717158277455	2	0
29	Fűzfői-séd	slow-flowing stream	47.0620536034907	18.0323762696994	2	0
30	Paloznak	marsh	46.9813784882151	17.9466214774899	2	0
31	Balatonboglár	canal	46.766221975944	17.6530318117254	1	19
32	Balatonmáriafürdő	canal	46.6938768408155	17.4427500353511	1	3

Table S2 A summary of the mean, SD, minimum and maximum values of the estimated detection and abundance covariates of the N-mixture models.

Detection covariates	Code	Mean	SD	Min	Max
Water temperature (°C)	WTEMP	10.88	2.10	5.49	15.41
Water depth (cm)	DEPTH	34.14	15.30	13.83	77.03
Percentage of emergent vegetation (%)	VEG	59.06	22.45	10.00	95.00
Julian date of the survey	DATE	-	-	95	113
Number of traps	TRAPS	8.81	5.03	4.00	18.00
**Abundance covariates**					
Nearest neighbour distance (km)	NNDIST	2.63	2.50	0.11	11.11
Waterbody size (km^2)	WATERBODY	0.36	0.61	0.0003	2.39
Urban land cover in the 250 m buffer zone (%)	URBAN250	26.84	31.01	0.00	100.00
Urban land cover in the 500 m buffer zone (%)	URBAN500	27.27	26.18	1.65	88.91
Urban land cover in the 1000 m buffer zone (%)	URBAN1000	28.23	19.25	2.20	73.19
Cropland cover in the 250 m buffer zone (%)	CROPLAND250	9.80	17.42	0.00	63.56
Cropland cover in the 500 m buffer zone (%)	CROPLAND500	11.93	15.82	0.00	59.00
Cropland cover in the 1000 m buffer zone (%)	CROPLAND1000	15.05	15.39	0.00	56.23
Wetland cover in the 250 m buffer zone (%)	WETLAND250	41.04	30.85	0.00	99.16
Wetland cover in the 500 m buffer zone (%)	WETLAND500	39.15	26.76	0.05	96.22
Wetland cover in the 1000 m buffer zone (%)	WETLAND1000	32.30	21.97	0.17	84.70
Forest and grassland cover in the 250 m buffer zone (%)	TERRSITES250	20.43	21.87	0.00	99.38
Forest and grassland cover in the 500 m buffer zone (%)	TERRSITES500	18.68	15.97	0.10	68.36
Forest and grassland cover in the 1000 m buffer zone (%)	TERRSITES1000	19.40	17.81	1.44	71.13
Distance to road (km)	DISTROAD	0.42	0.39	0.01	1.75
Distance to railway (km)	DISTRAILWAY	1.26	1.30	0.03	6.03

Table S3 Overview of Watanabe-Akaike Information Criterion (WAIC) and the estimated parameters for the abundance sub-models incorporating spatial autocorrelation and waterbody size. Models with ΔWAIC < 2 are considered the best ranking model. Estimates are provided with 95% Bayesian credible intervals (representing the 2.5th and 97.5th percentiles of the posterior distribution) and the proportion of the posterior distribution (*f*). Covariate relationships were considered important if their 95% Bayesian credible intervals did not overlap zero or if the proportion of the posterior distribution exceeded 90% (in bold). Water depth (DEPTH) was included as a detection covariate in each model.

Model	WAIC	ΔWAIC	Parameters	Mean	SD	2.5^{th}	97.5^{th}	*f*
1	282,240	0,000	Intercept	2.474	0.367	1.822	3.261	
			**NNDIST**	**-0.289**	**0.213**	**-0.706**	**0.131**	**91.235**
			Intercept	-1.563	0.419	-2.457	-0.817	
			DEPTH	-0.632	0.160	-0.957	-0.328	
2	282,441	0,201	Intercept	2.433	0.356	1.786	3.182	
			**WATERBODY**	**0.295**	**0.203**	**-0.102**	**0.693**	**92.722**
			Intercept	-1.519	0.404	-2.365	-0.777	
			DEPTH	-0.584	0.158	-0.904	-0.285	
3	282,293	0,053	Intercept	2.436	0.365	1.787	-0.805	
			**NNDIST**	**-0.317**	**0.215**	**-0.741**	**0.104**	**93.000**
			**WATERBODY**	**0.320**	**0.205**	**-0.080**	**0.723**	**94.134**
			Intercept	-1.534	0.414	-2.425	-0.805	
			DEPTH	-0.618	0.161	-0.946	-0.316	

Table S4: The estimated parameters for the abundance sub-models of URBAN, CROPLAND, WETLAND and TERRSITES in the 250, 500 and 1000 m buffer zones. Estimates are provided with 95% Bayesian credible intervals (representing the 2.5th and 97.5th percentiles of the posterior distribution) and the proportion of the posterior distribution (*f*). Covariate relationships were considered important if their 95% Bayesian credible intervals did not overlap zero or if the proportion of the posterior distribution exceeded 90% (in bold). Water depth (DEPTH) was included as a detection covariate in each model.

Model	Parameters	Mean	SD	2.5^{th}	97.5^{th}	*f*
1	Intercept	2.447	0.369	1.795	3.246	
	**WATERBODY**	**0.305**	**0.205**	**-0.096**	**0.709**	**93.22**
	NNDIST	-0.183	0.250	-0.672	0.307	76.88
	URBAN250	-0.259	0.237	-0.725	0.206	86.29
	Intercept	-1.566	0.419	-2.481	-0.822	
	**DEPTH**	**-0.622**	**0.161**	**-0.947**	**-0.317**	**100.00**
2	Intercept	2.428	0.361	1.785	3.201	
	**WATERBODY**	**0.299**	**0.204**	**-0.101**	**0.701**	**92.93**
	NNDIST	-0.106	0.247	-0.592	0.377	66.69
	**URBAN500**	**-0.417**	**0.238**	**-0.882**	**0.041**	**96.26**
	Intercept	-1.552	0.408	-2.426	-0.821	
	**DEPTH**	**-0.34**	**0.162**	**-0.962**	**-0.327**	**100.00**
3	Intercept	2.434	0.361	1.785	3.199	
	**WATERBODY**	**0.297**	**0.204**	**-0.102**	**0.701**	**92.69**
	NNDIST	-0.211	0.225	-0.653	0.233	82.66
	**URBAN1000**	**-0.344**	**0.212**	**-0.761**	**0.067**	**94.91**
	Intercept	-1.560	0.408	-2.429	-0.821	
	**DEPTH**	**-0.610**	**0.160**	**-0.936**	**-0.311**	**100.00**
4	Intercept	2.382	0.383	1.694	3.200	
	**WATERBODY**	**0.348**	**0.201**	**-0.044**	**0.744**	**95.89**
	**NNDIST**	**-0.743**	**0.255**	**-1.249**	**-0.248**	**100.00**
	**CROPLAND250**	**-0.958**	**0.303**	**-1.581**	**-0.394**	**100.00**
	Intercept	-1.619	0.435	-2.529	-0.840	
	**DEPTH**	**-0.575**	**0.160**	**-0.900**	**-0.272**	**100.00**
5	Intercept	2.394	0.364	1.744	3.172	
	**WATERBODY**	**0.336**	**0.203**	**-0.062**	**0.737**	**95.13**
	**NNDIST**	**-0.698**	**0.268**	**-1.231**	**-0.177**	**100.00**
	**CROPLAND500**	**-0.648**	**0.262**	**-1.174**	**-0.146**	**100.00**
	Intercept	-1.569	0.41	-2.437	-0.834	
	**DEPTH**	**-0.608**	**0.160**	**-0.933**	**-0.713**	**100.00**
6	Intercept	2.431	0.364	1.787	3.213	
	**WATERBODY**	**0.324**	**0.204**	**-0.075**	**0.729**	**94.45**
	**NNDIST**	**-0.399**	**0.255**	**-0.899**	**0.101**	**94.13**
	CROPLAND1000	-0.132	0.220	-0.563	0.300	72.68
	Intercept	-1.547	0.413	-2.423	-0.819	
	**DEPTH**	**-0.623**	**0.162**	**-0.951**	**-0.316**	**100.00**
7	Intercept	2.443	0.378	1.779	3.272	
	WATERBODY	0.186	0.213	-0.230	0.607	80.89
	NNDIST	-0.221	0.222	-0.653	0.215	84.02
	**WETLAND250**	**0.458**	**0.207**	**0.053**	**0.865**	**100.00**
	Intercept	-1.594	0.430	-2.532	-0.838	
	**DEPTH**	**-0.593**	**0.159**	**-0.919**	**-0.296**	**100.00**
8	Intercept	2.417	0.369	1.758	3.207	
	WATERBODY	0.222	0.207	-0.182	0.629	85.88
	NNDIST	-0.176	0.222	-0.613	0.262	78.66
	**WETLAND500**	**0.727**	**0.201**	**0.335**	**1.125**	**100.00**
	Intercept	-1.621	0.417	-2.509	-0.873	
	**DEPTH**	**-0.648**	**0.162**	**-0.981**	**-0.343**	**100.00**

9	Intercept	2.434	0.373	1.770	3.235	
	**WATERBODY**	**0.232**	**0.208**	**-0.174**	**0.640**	**86.95**
	**NNDIST**	**-0.238**	**0.220**	**-0.669**	**0.195**	**86.02**
	**WETLAND1000**	**0.549**	**0.198**	**0.161**	**0.937**	**100.00**
	Intercept	-1.606	0.424	-2.510	-0.854	
	**DEPTH**	**-0.637**	**0.161**	**-0.965**	**-0.333**	**100.00**
10	Intercept	2.442	0.362	1.792	3.211	
	**WATERBODY**	**0.374**	**0.222**	**-0.061**	**0.810**	**95.42**
	**NNDIST**	**-0.332**	**0.218**	**-0.758**	**0.094**	**93.73**
	TERRSITES250	0.131	0.207	-0.275	0.539	73.71
	Intercept	-1.556	0.411	-2.422	-0.818	
	**DEPTH**	**-0.627**	**0.161**	**-0.953**	**-0.320**	**100.00**
11	Intercept	2.445	0.366	1.788	3.221	
	**WATERBODY**	**0.271**	**0.209**	**-0.137**	**0.683**	**90.32**
	NNDIST	-0.252	0.222	-0.687	0.185	87.19
	TERRSITES500	-0.249	0.214	-0.671	0.168	87.78
	Intercept	-1.567	0.416	-2.437	-0.817	
	**DEPTH**	**-0.627**	**0.163**	**-0.958**	**-0.319**	**100.00**
12	Intercept	2.441	0.367	1.781	3.208	
	**WATERBODY**	**0.308**	**0.205**	**-0.093**	**0.714**	**93.37**
	NNDIST	-0.259	0.229	-0.709	0.190	87.07
	TERRSITES1000	-0.172	0.223	-0.612	0.261	78.02
	Intercept	-1.560	0.418	-2.416	-0.803	
	**DEPTH**	**-0.619**	**0.161**	**-0.945**	**-0.314**	**100.00**
